# Detection of Monocyte Subsets in the Bone Marrow of Patients With Acute Myeloid Leukemia and Its Clinical Significance

**DOI:** 10.1155/jimr/2554167

**Published:** 2025-12-15

**Authors:** Liangjun Zhang, Man Chen, Huixiu Zhong, Jingyuan Huang, Ziling Xu, Minggang Yin, Yi Li, Chengjian Cao

**Affiliations:** ^1^ Department of Laboratory Medicine, Zigong First People’s Hospital, Zigong City, Sichuan Province, China; ^2^ Department of Laboratory Medicine, HeBei Yanda Lu Daopei Hospital, Yanda City, HeBei Province, China; ^3^ Department of Laboratory Medicine, Zigong Fourth People’s Hospital, Zigong City, Sichuan Province, China, zg120.cn; ^4^ Department of Laboratory Medicine, West China Hospital, Sichuan University, Chengdu, China, scu.edu.cn

**Keywords:** acute myeloid leukemia, classical monocytes, intermediate monocytes, monocyte subsets, prognosis

## Abstract

**Background:**

The bone marrow (BM) microenvironment plays a crucial role in acute myeloid leukemia (AML), but the distribution and clinical significance of monocyte subsets within this compartment remain poorly characterized. This study aimed to investigate the composition of BM monocyte subpopulations and their relationship with systemic immunity and clinical outcomes in AML patients.

**Methods:**

We collected BM samples from 98 AML patients (including 23 newly diagnosed, 28 nonremission, and 47 complete remission [CR] cases) and 23 healthy controls (HCs). Using flow cytometry, we analyzed monocyte subsets (classical, intermediate, nonclassical) and monocytic myeloid‐derived suppressor cells (m‐MDSCs) in BM, along with T lymphocyte subsets in peripheral blood. Survival analysis was performed with 1‐year follow‐up data.

**Results:**

Both the proportion of intermediate monocytes and m‐MDSCs among total monocytes were significantly elevated in newly diagnosed AML patients compared to those in HCs (*p*  = 0.019 and *p*  = 0.003, respectively) and CR (*p* = 0.003 and *p* = 0.037, respectively). This elevation was followed by a gradual decrease from diagnosis to remission. Multivariate Cox regression identified intermediate monocyte percentage as an independent prognostic factor (HR = 4.170, *p* = 0.034). Kaplan–Meier analysis confirmed that higher intermediate monocyte levels predicted shorter overall survival (OS) (*p* = 0.031) and leukemia‐free survival (LFS) (*p* = 0.028). Importantly, negative correlations were observed between BM blasts and peripheral blood T‐cell percentage (*r* = −0.467, *p* = 0.005) and CD8^+^ T cells (*r* = −0.504, *p* = 0.002), and between intermediate monocytes among total monocytes and total T‐cell percentage (*r* = −0.475, *p* = 0.004).

**Conclusions:**

BM monocyte subsets, particularly intermediate monocytes, serve as significant indicators of disease progression and survival in AML. Their correlation with peripheral T‐cell immunity suggests their potential role in modulating antileukemic immune responses. These findings highlight the prognostic value of BM monocyte profiling and provide insights for developing novel immunotherapeutic strategies in AML.

## 1. Introduction

Acute myeloid leukemia (AML) is a hematologic malignancy characterized by marked heterogeneity, rapid onset, and aggressive progression. It arises from the abnormal proliferation of immature myeloid progenitor cells within the bone marrow (BM) [1]. Among all forms of leukemia, AML accounts for the highest proportion of cases (~35% of total diagnoses) and exhibits the highest mortality rate, with a 5‐year survival rate of only about 30% [2]. In recent years, growing evidence has revealed that the tumor microenvironment in AML exerts immunosuppressive effects, underscoring a close relationship between disease progression and immune dysregulation. Consequently, elucidating alterations in the tumor microenvironment during AML initiation and development has become a pivotal research direction for developing novel therapeutic strategies.

Monocytes, which originate from hematopoietic stem cells, serve as precursors to macrophages and dendritic cells. They participate in innate and adaptive immune responses by presenting antigenic determinants to lymphocytes and promoting antigen‐specific immunity. Studies have shown that BM‐derived mesenchymal stromal cells can indirectly suppress the functions of T cells and natural killer (NK) cells by inducing the generation of monocytic myeloid‐derived suppressor cells (m‐MDSCs) from monocytes. Furthermore, m‐MDSCs exhibit elevated expression of the inhibitory ligands PD‐L1 and CD155 [3]. Impaired differentiation and maturation of monocytes have also been observed in patients with myelodysplastic syndrome (MDS), and these defects tend to accumulate as the disease advances [3]. Based on surface expression of CD14 and CD16, monocytes are categorized into three subsets: classical, intermediate, and nonclassical monocytes. In AML, classical monocytes have been reported to display elevated autophagy levels, which correlate with their expanded proportion; hypoxia has been identified as a key driver of this phenomenon [4].

MDSCs represent a heterogeneous population of immature myeloid cells arrested at various stages of differentiation. These cells acquire potent immunosuppressive properties [5] and consist mainly of myeloid progenitors and immature granulocytic or monocytic cells. They accumulate during cancer progression and facilitate immune escape via multiple mechanisms [6]. Significant alterations in immune cell infiltration (including T cells, B cells, NK cells, NKT cells, and anti‐inflammatory immune cells) have been documented in the BM of AML patients compared with healthy individuals [7]. MDSC frequencies in peripheral blood are substantially higher in AML patients than in healthy controls (HCs) and are notably elevated at diagnosis compared to post‐chemotherapy remission [8]. Although MDSCs promote tumor progression largely by suppressing T‐cell activity, their precise role in AML pathogenesis remains incompletely defined.

The BM serves as the primary site of origin and key pathological niche in myeloid malignancies, where neoplastic clones emerge, expand, and engage in aberrant differentiation and cellular interaction. However, most previous studies have been centered on peripheral blood, with relatively limited investigation devoted to BM specimens. We thus hypothesized that shifts in monocyte subsets in the BM may more accurately reflect core disease‐specific biology, being less affected by extrinsic nonhematologic factors, such as infection or systemic inflammation, than those in peripheral blood. Based on this premise, this study was designed to explore the composition and clinical relevance of monocyte subsets in BM samples from AML patients.

## 2. Patients and Methods

### 2.1. Sample Size Calculation

An a priori sample size calculation was conducted using 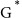
Power software (version 3.1.9.7). The calculation was based on detecting a significant difference in the percentage of intermediate monocytes among the four groups (newly diagnosed AML, nonremission AML, remission AML, and HCs). We selected one‐way analysis of variance (ANOVA) as the statistical test. The parameters were set as follows: an alpha level of 0.05, a statistical power of 0.80, and a large effect size (*f* = 0.4). This effect size was chosen based on the findings of a previous study by Hu et al. [9] and was considered clinically significant in our pilot data. The calculation indicated that a total sample size of 76 participants was required. With four groups, this translates to a minimum of 19 participants per group. To account for potential data loss due to sample processing issues or technical failures in flow cytometry, we aimed to enroll 23 participants per group, resulting in a total target sample size of 100.

### 2.2. Patients

BM samples were obtained from inpatients at Zigong First People’s Hospital. Written informed consent was obtained from all participants, and the study protocol received approval from the hospital’s Ethics Committee (Approval Number 03202024). A total of 98 patients with AML were enrolled, along with 23 HCs. All patients were treated with a combination of azacytidine and venetoclax and were categorized into three groups based on treatment response: 23 newly diagnosed patients, 28 with incomplete remission after chemotherapy, and 47 who achieved complete remission (CR). These patients were admitted to Zigong First People’s Hospital between 2023 and 2024. CR was defined according to the International Working Group recommendations for diagnosis and response criteria in AML [10], requiring normalization of blood counts and BM morphology, along with the absence of all signs of leukemia for at least 4 weeks. Patients with a history of other hematological or autoimmune diseases were excluded from the study. Among the 23 newly diagnosed AML patients followed for 1 year, 13 died and 10 survived. All deceased patients had either experienced relapse or failed to achieve remission following chemotherapy, whereas all surviving patients maintained CR.

### 2.3. Flow Cytometric Analysis

We followed the methods of Liangjun Zhang et al. [11]. BM samples anticoagulated with EDTA were diluted 1:1 with phosphate‐buffered saline (PBS; Beckman Coulter [BC], California, USA). Ficoll separation solution (BC) was placed in a centrifuge tube, and the diluted BM–PBS mixture was carefully layered on top. Centrifugation was then performed at 500 × *g* for 20 min to isolate BM mononuclear cells (BMMCs). The BMMC layer was carefully collected, transferred to a new tube, and washed twice with PBS by centrifugation at 1500 rpm for 10 min. After the final wash, the cell pellet was resuspended, and the BMMC concentration was adjusted to 1 × 10^6^/mL. Aliquots of 100 µL cell suspension were incubated in the dark at room temperature (20–25°C) for 15 min with the following antibody panel: HLADR‐FITC, CD15‐PE, CD16‐ECD, CD11b‐APC, CD33‐PC5.5, CD45‐PC7, and CD14‐APC750 (all from BC, USA). Following incubation, cells were washed with 1 mL of PBS and centrifuged at 500 × *g* for 5 min. The supernatant was discarded, and the cells were resuspended in 200 µL of PBS for acquisition. Flow cytometry was performed on a BC NAVIOS instrument, and data from 200,000 events per sample were collected and analyzed using Kaluza Analysis Software v2.1 (BC). All experiments were conducted in accordance with relevant guidelines and regulations.

The following describes the processing methods for peripheral blood. First, 20 µL of Beckman multicolor antibody reagent (CD45‐FITC, CD4‐PE, CD8‐ECD, and CD3‐PC5.5) was added to the test tube, followed by the precise addition of 100 µL of well‐mixed anticoagulated whole blood. Then, 100 µL of thoroughly vortexed quantitative microspheres (Flow‐Count Fluorospheres, BC) was immediately added. The mixture was gently vortexed and incubated at room temperature, protected from light, for 15–30 min. Subsequently, a lysing reagent (BC) was directly added to lyse the red blood cells, followed by another incubation at room temperature, protected from light, for 10–15 min until the solution becomes clear. Upon completion of this step, no washing was required; the mixture was vortexed to ensure homogeneity before direct acquisition on the flow cytometer. Finally, data were acquired on the flow cytometer, ensuring that a sufficient number of microspheres (>2500 events) were collected. The absolute counts of each lymphocyte subset were automatically calculated by the Kaluza Analysis software based on the known concentration of the microspheres and the number of acquired cell and microsphere events.

**Figure 1:**
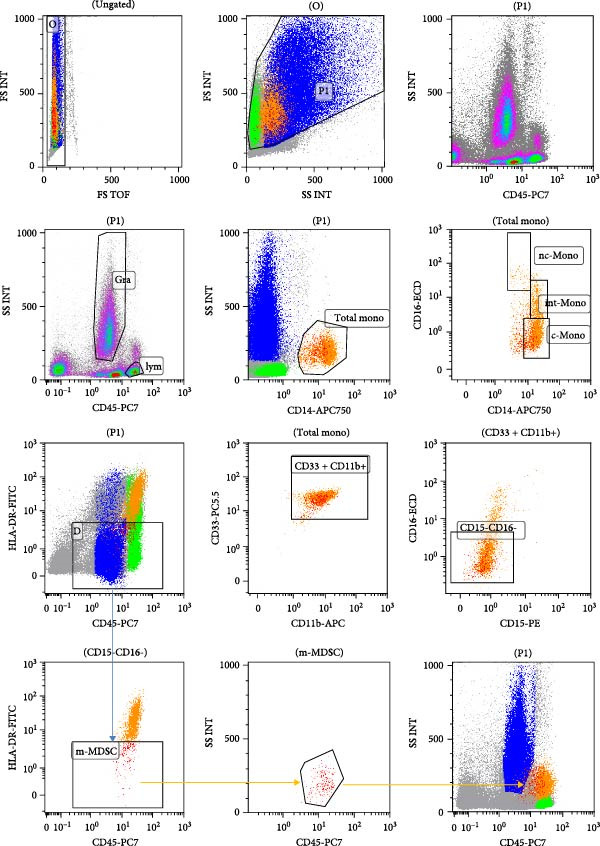
Gating strategy for monocyte subsets in bone marrow samples from HCs.

**Figure 2:**
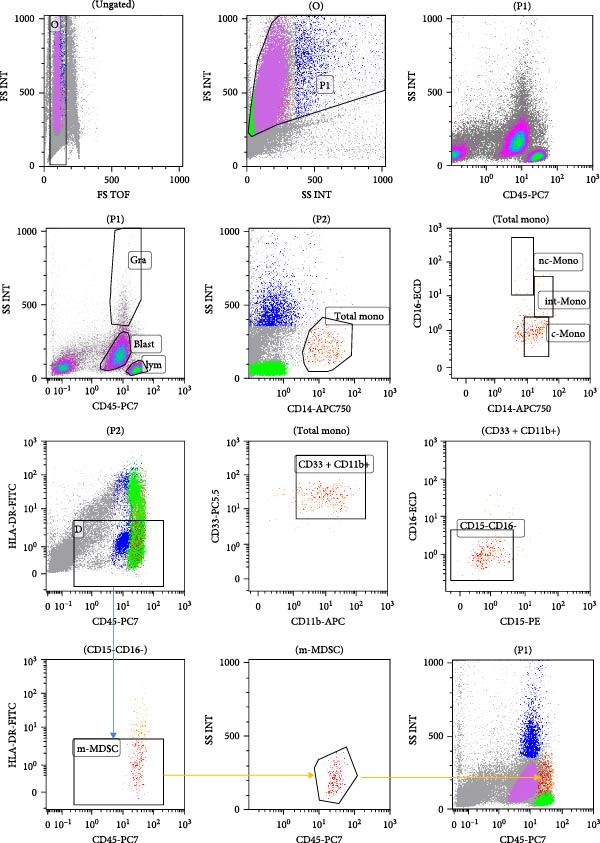
Gating strategy for monocyte subsets in bone marrow samples from AML patient.

The analysis of monocyte subset cells was carried out in the following steps successively, as shown in Figure 1. First, doublets were excluded based on forward scatter time‐of‐flight (FS TOF) versus forward scatter integral (FS INT). Subsequently, all nucleated cells within the BM were gated using FS and side scatter (SSC), excluding debris. From this population, granulocytes (blue) and lymphocytes (green) were identified on a CD45 versus SSC plot. Putative blast cells were initially gated according to their characteristic CD45dim SSC low immunophenotype and were subsequently confirmed using a separate antibody tube containing CD34 and CD117 markers. The CD34^+^/CD117^+^ population within the CD45low SSC low gate was definitively identified as leukemic blasts and excluded from downstream analysis (Figure 2). Total monocytes were then gated using CD14 versus SSC, and monocyte subsets—classical (CD14^++^CD16^−^), intermediate (CD14^++^CD16^+^), and nonclassical (CD14^+^CD16^++^)—were further resolved based on their CD14 and CD16 expression. The total monocyte was further refined by selecting CD33^+^CD11b^+^ cells on a CD33/CD11b plot. These cells were then subjected to gating for CD16^−^CD15^−^ monocytic cells. Finally, to identify mMDSCs, a CD45 versus HLA‐DR plot was used, wherein HLA‐DR‐negative cells were gated, with the HLA‐DR negativity defined using granulocytes and lymphocytes as reference populations. The following cell subtypes were analyzed in this study: m‐MDSC cells (CD14^+^CD33^+^CD11b^+^CD15^−^CD16^−^HLADR^-^), classical monocyte (CD14^++^CD16^−^), intermediate monocyte (CD14^++−^CD16^+^), and non‐classical monocyte (CD14^+−^CD16^++^).

**Figure 3:**
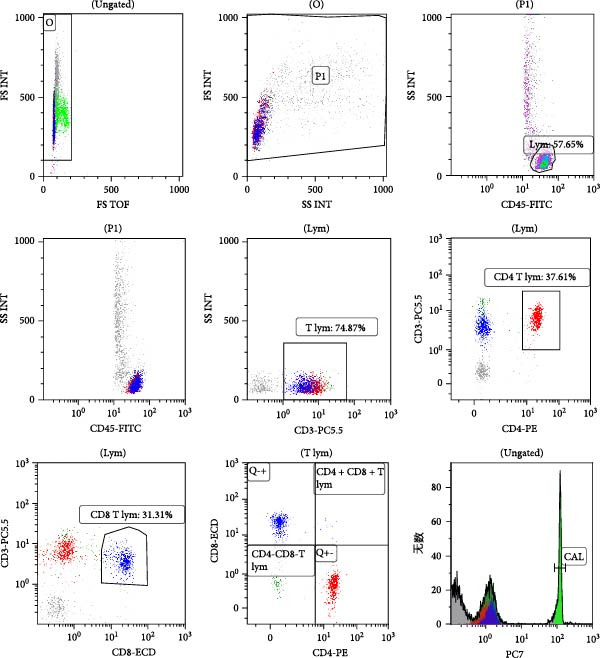
Gating strategy for lymphocyte subsets in peripheral blood.

A standardized gating strategy was employed for lymphocyte percentages and absolute counting (Figure 3). Initial data acquisition involved the elimination of cell doublets by gating on single cells using a plot of FS TOF versus FS INT. Subsequently, a primary gate was set on the scatter plot of FSC versus SSC to identify the intact cell population while excluding cellular debris. A sequential gating strategy was then applied: lymphocytes were precisely identified from the primary gate based on bright CD45 expression and low SSC characteristics. From this lymphocyte gate, total T lymphocytes were delineated based on positive CD3 expression. Further subtyping of T lymphocytes was performed within the lymphocyte gate using CD4 versus CD3 and CD8 versus CD3 dot plots, enabling the discrimination of CD4^+^ single‐positive, CD8^+^ single‐positive, CD4^+^CD8^+^ double‐positive, and CD4^−^CD8^−^ double‐negative T‐cell subsets. For the determination of absolute cell counts, quantitative microspheres were gated and enumerated using a histogram of the PC7 fluorescence channel.

### 2.4. Statistical Analyses

**Figure 4:**
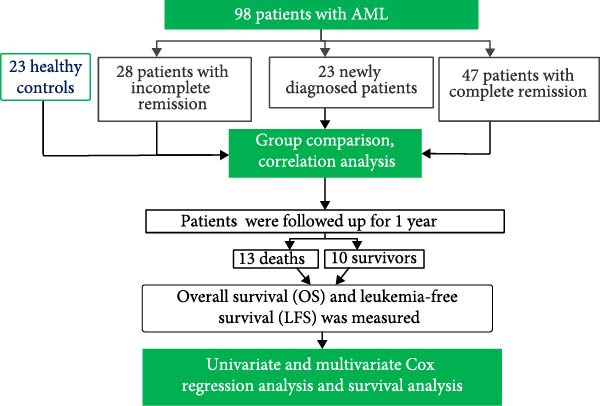
Technology roadmap of the study.

Flow cytometric data were analyzed using Kaluza software. Subsequent statistical analysis and visualization were performed with GraphPad Prism 5.01. As all cellular data exhibited nonnormal distributions, results are presented as median with interquartile range for each group. For comparisons between two groups of nonnormally distributed lymphocyte subsets, the Mann–Whitney *U* test was applied, while the Kruskal–Wallis test was used for multigroup comparisons. Correlation analyses were conducted using Pearson’s correlation test. Overall survival (OS) was defined as the time from diagnosis to death from any cause. Leukemia‐free survival (LFS) was measured from the date of CR achievement to either relapse at the last follow‐up or death without prior relapse. Univariate and multivariate Cox regression analyses were performed to identify independent prognostic risk factors. Survival curves were generated using the Kaplan–Meier method and compared with the log‐rank test. A two‐sided *p*‐value <0.05 was considered statistically significant. All statistical analyses were carried out using SPSS software (version 25.0; IBM Corp.). The overall technical workflow of this study is summarized in Figure 4.

## 3. Results

### 3.1. Clinical Features of Patients With AML and HC

Based on their response to chemotherapy, AML patients were divided into three groups (newly diagnosed, incomplete remission, and CR). No significant differences in age or gender were found among these patient groups and the HC group. (*p* > 0.05). The disease was divided into two major groups according to FAB classification (M_1/2_ and M_4/5_), and there was no statistical difference between the three groups (*p* > 0.05). The NRS2002 score, which included the disease score and the impaired nutrition score and was published by the European Society for Clinical Nutrition and Metabolism (ESPEN) [12], is a reliable nutritional risk screening indicator for patients with AML. The Padua score was constructed at the University of Padua in Italy on the basis of reviewing the previous risk assessment model of venous thrombosis (VET) in internal medicine inpatients [13] and was recommended by the guideline [14] to evaluate the risk of VET in internal medicine inpatients. The scale includes 11 risk factors, such as active tumor, thrombotic history, immobilization, and age. Each factor is scored with 1–3 points. The higher the total score, the higher the risk of thrombosis. According to the total score, the risk of thrombosis was divided into two grades (VET risk scale): <4 was classified as low risk, and ≥4 was classified as high risk. Genetic risk stratification was based on the 2022 European LeukemiaNet (ELN) criteria [15]. There were no differences in disease score, impaired nutrition score, NRS2002 total score, Padua score, VET risk scale, and ELN 2022 among the three groups (*p* > 0.05). The demographic characteristics of AML are shown in Table 1.

**Table 1:** Demographic characteristics of AML and HC.

Variables	HCs (*n* = 23)	Newly diagnosed (*n* = 23)	Incomplete remission (*n* = 28)	Complete remission (*n* = 47)	*p*
Sex, *n* (%)	—	—	—	—	0.378
Male	11 (47)	10 (43)	9 (32)	27 (57)	—
Female	12 (52)	13 (57)	19 (68)	20 (43)	—
Age, median (Q1, Q3)	58.63 (53, 75)	64 (60.5, 78)	62 (57, 68)	58 (54, 64)	0.216
Diagnose type, *n* (%)	—	—	—	—	0.353
AML‐M_1/2_	—	11 (48)	14 (50)	31 (66)	—
AML‐M_4/5_	—	12 (52)	14 (50)	16 (34)	—
Disease score, *n* (%)	—	—	—	—	0.061
0	—	6 (26)	1 (4)	4 (9)	—
1	—	3 (13)	1 (4)	1 (2)	—
2	—	14 (61)	25 (89)	40 (85)	—
3	—	0 (0)	1 (3)	2 (4)	—
Impaired nutrition score, *n* (%)	—	—	—	—	0.350
0	—	18 (83)	24 (86)	43 (91)	—
1	—	1 (4)	2 (7)	0 (0)	—
2	—	0 (0)	1 (4)	1 (2)	—
3	—	4 (13)	1 (4)	3 (6)	—
NRS2002 total score, median (Q1, Q3)	—	2 (1,3)	2 (2,3)	2 (2,2)	0.974
Padua score, median (Q1, Q3)	—	1 (0.5,5)	3.5 (3,4)	3 (3,4)	0.204
VET risk scale, *n* (%)	—	—	—	—	0.127
Low risk	—	13 (57)	15 (54)	35 (74)	—
High risk	—	10 (43)	13 (46)	12 (26)	—
ELN 2022	—	—	—	—	0.066
Favorable	—	4 (17)	12 (43)	21 (45)	—
Intermediate	—	15 (65)	8 (29)	17 (36)	—
Unfavorable	—	4 (17)	8 (29)	9 (19)	—

### 3.2. Percentage of Monocyte Subsets in the BM of Patients With AML During Different States

Significant differences were found among the newly diagnosed, incomplete remission, CR, and HC groups in the percentages of blast cells/BMMCs, total monocytes/BMMCs, intermediate monocytes/total monocytes, and m‐MDSC/total monocytes. (*p*  < 0.001, *p* = 0.002, *p* = 0.004, and *p* = 0.002, respectively) (Table 2); following chemotherapy, the proportions of both intermediate monocytes and m‐MDSCs within the total monocytes exhibited a declining trend from newly diagnosed to CR. Notably, these proportions were significantly different not only between the HCs and the newly diagnosed group (*p* = 0.019 and *p* = 0.003, respectively), but also between the newly diagnosed and CR groups (*p* = 0.003, and *p* = 0.037, respectively) (Figure 5). No difference was observed in the percentage of m‐MDSC/BMMCs, classical monocytes/total monocytes, and nonclassical monocytes/total monocytes between the four groups (*p* > 0.05).

Figure 5Monocyte subsets in BM samples from healthy controls and patients with AML across different disease stages. (A) Percentage of intermediate monocytes among total monocytes across the four groups. (B) Percentage of m‐MDSCs among total monocytes across the four groups.  ^∗^ *p* < 0.05 and  ^∗∗^ *p* < 0.01.

**Figure:**
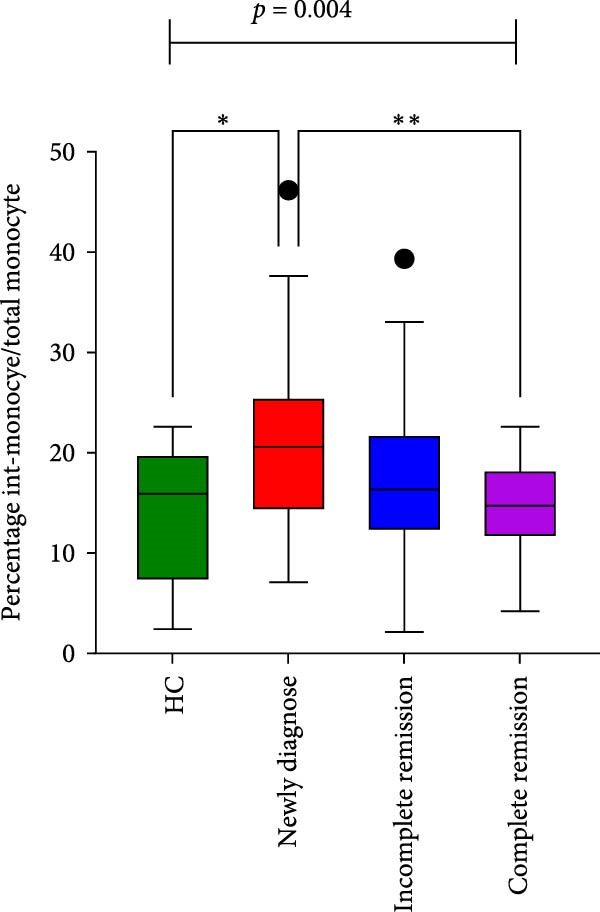
(A)

**Figure:**
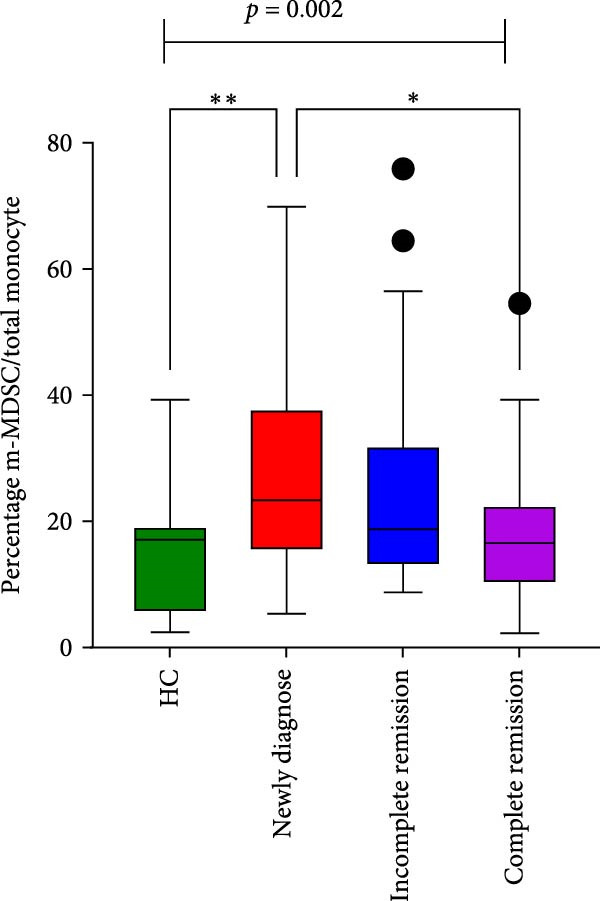
(B)

**Table 2:** Percentage of the monocyte subsets in the BM of patients with AML and HC.

Variables	HCs (*n* = 23)	Newly diagnosed (*n* = 23)	Incomplete remission (*n* = 28)	Complete remission (*n* = 47)	*p*
%Blast/BMMCs, median (Q1,Q3)	—	71 (54, 78)	12.79 (3.39, 35.5)	0 (0, 0)	***<0.001***
%Total monocyte/BMMCs, median (Q1,Q3)	3.07 (2.21, 4.02)	1.28 (0.36, 3.33)	3.32 (2.2, 6.16)	4.34 (2.84, 5.1)	***0.002***
%m‐MDSC/BMMCs, median (Q1,Q3)	0.38 (0.13, 0.77)	0.14 (0.03, 0.81)	0.22 (0.07, 0.4)	0.34 (0.23, 0.86)	0.222
%c‐Monocyte/total monocyte, median (Q1,Q3)	69.95 (65.87, 76.46)	66.92 (52.33, 71.44)	66.75 (48.72, 74.57)	69.39 (62.27, 76.98)	0.085
%int‐Monocye/total monocyte, median (Q1,Q3)	15.95 (7.36, 19.48)	20.6 (16.24, 24.88)	16.36 (12.2, 21.02)	14.74 (11.54, 18.07)	***0.004***
%nc‐Monocye/total monocyte, median (Q1,Q3)	13.17 (7.87, 16.39)	14.33 (7.09, 23.28)	13.28 (7.41, 22.2)	12.57 (8.78, 16.83)	0.966
%m‐MDSC/total monocyte, median (Q1,Q3)	17.13 (5.4, 19.43)	23.39 (16.81, 33.96)	18.78 (12.94, 27.94)	16.58 (9.99, 22.42)	***0.002***

*Note: p*‐Values <0.05 are shown in bold and italic.

### 3.3. Dysregulation of Peripheral Blood T‐Cell Subsets in AML and Their Correlation With BM Monocyte Subsets

The aforementioned results demonstrate significant associations between monocyte subset distribution in the BM microenvironment and patient prognosis. To investigate the potential link between the host’s systemic immune status and the intrinsic characteristics of the myeloid malignancy, we extended our analysis to lymphocyte subsets in peripheral blood. Our investigation revealed the alterations in both the proportion and absolute numbers of T‐cell subsets across different disease states in AML patients compared to HCs. The absolute counts of total lymphocytes, T cells (CD3^+^), CD4^+^ T cells, CD8^+^ T cells, and CD4^−^CD8^−^ T cells showed a progressive and statistically significant decline from HCs to newly diagnosed AML patients and further to nonremission patients (*p* < 0.001 for all comparisons; Table 3). Analysis of relative frequencies also demonstrated significant differences among the four groups. The percentage of total T cells (CD^3+^) among lymphocytes varied significantly across groups (*p* < 0.001), with similar significant variations observed in the percentages of CD4^+^ T cells (*p* = 0.006) and CD8^+^T cells (*p* < 0.001) within the total lymphocytes population.

**Table 3:** Number and percentage of the T lymphocyte cell subsets in the PB of patients with AML and HCs.

Variables	HC (*n* = 23)	Newly diagnosed (*n* = 23)	Incomplete remission (*n* = 28)	Complete remission (*n* = 47)	*p*
Number of lymphocytes/μL, median (Q1,Q3)	2227 (1778.5, 2566.5)	1465 (1255.75, 1683.25)	807 (531, 1087)	869.5 (518.25, 1019.75)	<0.001
%Total T cells/lymphocytes, median (Q1,Q3)	72.84 (62.61, 76.66)	67.21 (64.64, 71.59)	85.07 (82.22, 93.54)	83.42 (78.84, 87.72)	<0.001
Number of T cells/μL, median (Q1,Q3)	1601 (1118, 1775.5)	1035.53 (785.4, 1235.62)	678.44 (437.22, 938.14)	694.4 (455.69, 822.81)	<0.001
% CD4 T cells/lymphocytes, mean ± SD	37.5 ± 11.19	43.28 ± 12.26	47.89 ± 11.38	36.81 ± 13.65	0.006
Number of CD4 T cells/μL, median (Q1,Q3)	694 (572.5, 1021)	621.15 (371.34, 894.21)	403.17 (243.55, 562.59)	333.6 (198.86, 425.47)	<0.001
% CD8 T cells/lymphocytes, mean ± SD	23.09 ± 7.59	20.7 ± 6.96	30.38 ± 8.63	38.76 ± 9.81	<0.001
Number of CD8 T cells/μL, median (Q1,Q3)	497 (301, 690.5)	255.92 (198.6, 464.63)	213.57 (151.65, 326.02)	285.87 (198.19, 419.95)	<0.001
% CD4^−^ CD8^−^ T cells/lymphocytes, median (Q1,Q3)	9.59 (8.16, 10.91)	5.93 (3.69, 8.21)	5.25 (3.7, 10.54)	6.6 (3.18, 9.71)	0.070
Number of CD4^−^ CD8^−^ T cells/μL, median (Q1,Q3)	136 (89.5, 173.5)	65.39 (44.31, 122.71)	46.83 (28.62, 63.52)	42.84 (18.87, 76.19)	<0.001
% CD4^+^ CD8^+^ T cells/lymphocytes, median (Q1,Q3)	0.25 (0.15, 0.4)	0.63 (0.29, 0.81)	0.39 (0.28, 0.6)	0.45 (0.24, 0.72)	0.269
Number of CD4^+^ CD8^+^ T cells/μL, median (Q1,Q3)	4 (2, 8)	6.89 (3.74, 14.15)	3.33 (1.95, 4.36)	3.38 (2.19, 5.34)	0.114

**Figure 6:**
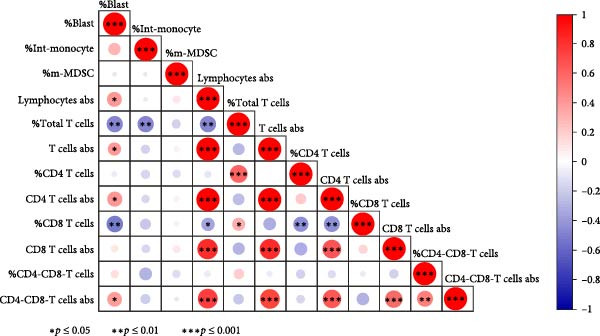
Heat map of the correlation values between monocyte subsets in the BM and lymphocyte subsets in the PB samples. Red and blue colors represent positive and negative correlations, respectively.

**Figure 8:**
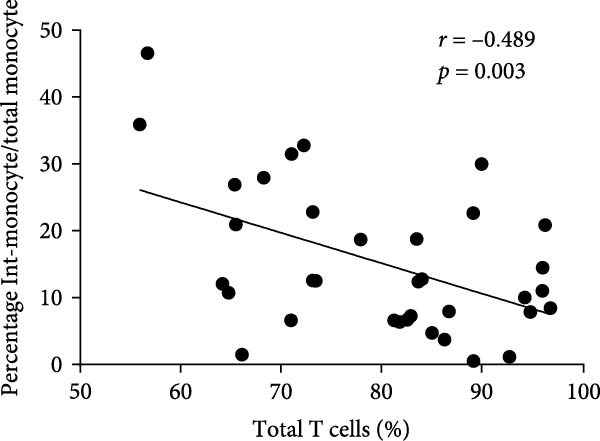
Correlation between the percentages of intermediate monocytes and total T cells in the PB samples.

In order to further explore the association between monocyte subsets in BM and T lymphocyte subsets in peripheral blood, we conducted a correlation analysis (Figure 6). The percentage of blasts had a strong negative correlation with the percentage of total T cells and CD8 T cells in the PB samples (*r* = 0.467, *p* = 0.005; *r* = 0.504, *p* = 0.002, respectively) (Figure 7). The percentage of intermediate monocytes showed a correlation with the percentage of total T cells in the PB samples (*r* = 0.475, *p* = 0.004) (Figure 8).

Figure 7(A) Correlation between the percentage of blast and total T cell in the PB samples. (B) Correlation between the percentage of blasts and CD8 T cells in the PB samples.

**Figure:**
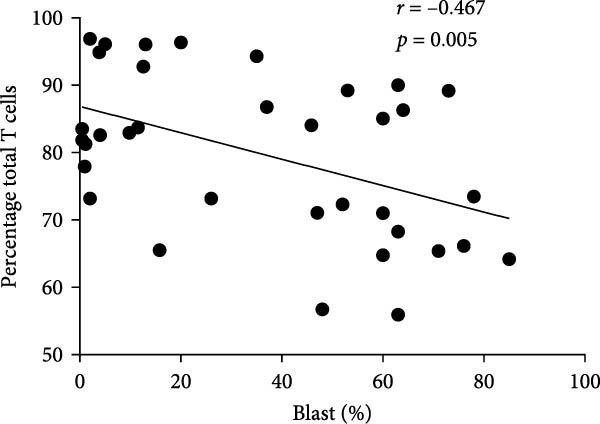
(A)

**Figure:**
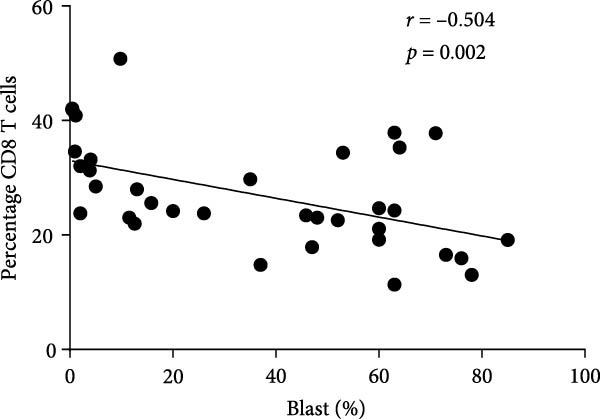
(B)

### 3.4. Univariate and Multivariate Cox Regression Analysis of Newly Diagnosed Patients With AML

Univariate and multivariate Cox regression analyses were performed to identify factors associated with OS in newly diagnosed AML patients. In the univariate analysis, three variables demonstrated statistically significant associations with OS: Padua score (HR = 1.434, 95% CI: 1.054–1.951, *p* = 0.022), ELN 2022 risk classification (HR = 2.379, 95% CI: 0.980–5.773, *p* = 0.035), and percentage of intermediate monocytes in total BM monocytes (HR = 1.065, 95% CI: 1.003–1.132, *p* = 0.039). Variables that were significant in univariate analysis (*p*  < 0.05) along with clinically relevant factors were included in the multivariate Cox regression model. The multivariate analysis revealed that two factors remained independent prognostic indicators for OS: ELN 2022 risk classification (HR = 3.264, 95% CI: 1.219–8.744, *p* = 0.019) and percentage of intermediate monocytes in total BM monocytes (HR = 4.170, 95% CI: 1.114–15.602, *p* = 0.034). Notably, the percentage of intermediate monocytes demonstrated a substantially increased hazard ratio in the multivariate model (4.170) compared to the univariate analysis (1.065), suggesting its strong independent prognostic value when adjusted for other factors. Other variables, including demographic factors (sex, age), clinical scores (disease score, impaired nutrition score, NRS2002 total score, VET risk scale), and various BM and peripheral blood immune cell populations, did not show statistically significant associations with OS in either univariate or multivariate analyses (Table 4).

**Table 4:** Univariate and multivariate Cox regression analysis of OS in newly diagnosed AML patients.

Variables	Univariate analysis			Multivariate analysis		
	HR	95% CI	*p*	HR	95% CI	*p*
Sex	0.596	0.199–1.783	0.354	—	—	—
Age	1.048	0.992–1.108	0.097	—	—	—
Diagnose type	0.819	0.274–2.445	0.721	—	—	—
Disease score	1.343	0.673–2.678	0.403	—	—	—
Impaired nutrition score	1.196	0.789–1.814	0.400	—	—	—
NRS2002 total score	1.397	0.988–1.974	0.058	—	—	—
Padua score	1.434	1.054–1.951	***0.022***	1.224	0.910–1.646	0.182
VET risk scale	2.305	0.769–6.915	0.136	—	—	—
ELN 2022	2.379	0.980–5.773	***0.035***	3.264	1.219–8.744	***0.019***
In BM sample						
%Blast/BMMCs	1.007	0.978–1.038	0.632	—	—	—
%Total monocyte/BMMCs	0.991	0.902–1.088	0.845	—	—	—
%m‐MDSC/BMMCs	0.695	0.339–1.424	0.320	—	—	—
%c‐Monocyte/total monocyte	0.952	0.912–0.993	0.062	—	—	—
%int‐Monocye/total monocyte	1.065	1.003–1.132	***0.039***	4.170	1.114–15.602	***0.034***
%nc‐Monocye/total monocyte	1.016	0.971–1.064	0.487	—	—	—
%m‐MDSC/total monocyte	0.974	0.939–1.011	0.168	—	—	—
In PB sample						
Number of lymphocytes	1.002	0.999–1.004	0.198	—	—	—
%Total T cells	0.994	0.877–1.128	0.924	—	—	—
Number of T cells	0.999	0.998–1.000	0.181	—	—	—
%CD4 T cells	0.970	0.895–1.050	0.448	—	—	—
Number of CD4 T cells	0.999	0.997–1.000	0.151	—	—	—
%CD8 T cells	1.077	0.973–1.192	0.152	—	—	—
Number of CD8 T cells	0.999	0.995–1.002	0.451	—	—	—
%CD4^−^CD8^−^ T cells	0.887	0.737–1.066	0.200	—	—	—
Number of CD4^−^CD8^−^ T cells	0.994	0.987–1.002	0.131	—	—	—
%CD4^+^CD8^+^ T cells	1.214	0.250–5.880	0.810	—	—	—
Number of CD4^+^CD8^+^ T cells	0.990	0.916–1.070	0.801	—	—	—

*Note: p*‐Values <0.05 are shown in bold and italic.

### 3.5. Survival Analysis for Intermediate Monocytes in Patients With AML

Based on Kaplan–Meier survival analysis, patients stratified by the median percentage of intermediate monocytes (%int‐monocyte/total monocyte) in the BM at new diagnosis exhibited significantly distinct survival outcomes. Patients in the group with more than 20% intermediate monocytes had significantly shorter OS compared to those with less than 20% (*p* = 0.031). Similarly, LFS was also significantly lower in the high intermediate monocyte group (>20%) than in the low group (*p* = 0.028) (Figure 9).

Figure 9(A) Overall survival and (B) leukemia‐free survival rates in the high and low intermediate monocyte groups.

**Figure:**
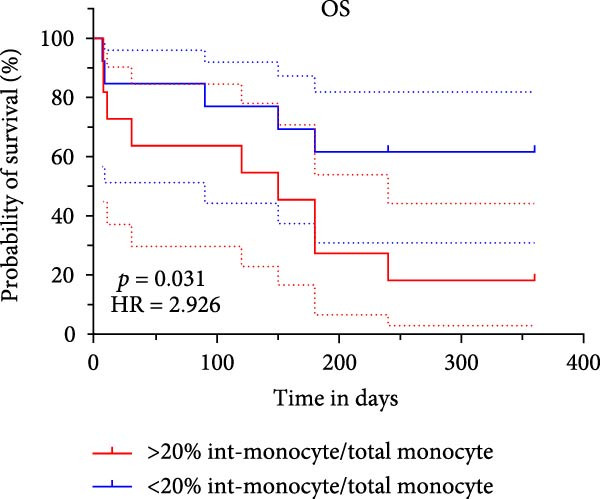
(A)

**Figure:**
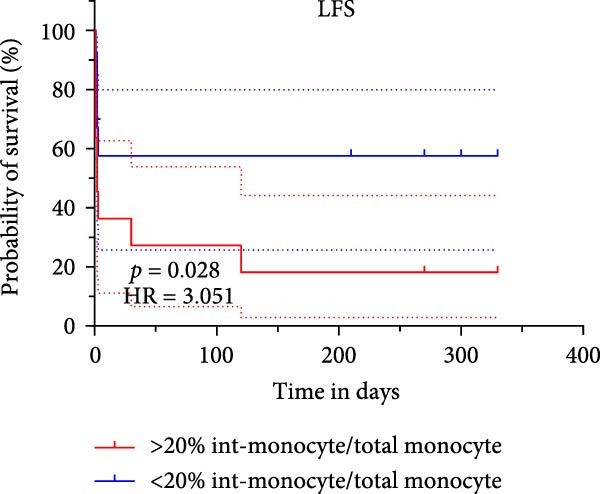
(B)

## 4. Discussion

AML represents a common and aggressive adult leukemia with a high mortality rate. Although conventional treatments such as chemotherapy and hematopoietic stem cell transplantation remain standard, outcomes remain unsatisfactory, with only approximately one‐third of patients achieving sustained remission. Relapsed disease continues to pose a substantial risk of mortality, highlighting the urgent need for more effective therapeutic strategies. In this context, immunotherapy has emerged as a promising approach for AML treatment [16].

MDSCs, a heterogeneous population of immature myeloid cells with immunosuppressive activity [17], have been implicated in tumor progression. These cells accumulate in cancer patients and facilitate immune escape through multiple mechanisms, thereby undermining anti‐tumor immunity. In AML, several studies have reported that MDSC levels are significantly elevated compared to HCs and are markedly higher at diagnosis than in patients who achieve CR after chemotherapy [8]. Furthermore, granulocytic MDSC levels in peripheral blood were shown to be higher in AML‐M2 than in other subtypes, while total MDSC and m‐MDSC levels were more prominent in early‐stage AML‐M3 patients [18]. However, most existing studies have focused on peripheral blood, and data on MDSCs within the BM microenvironment remain limited. In this study, we observed that the proportion of m‐MDSCs among total monocytes was significantly elevated in AML patients compared to HCs. Notably, this frequency exhibited a decline from newly diagnosed patients to those in CR, suggesting a potential role for m‐MDSCs in disease persistence and treatment response. These findings underscore the clinical relevance of BM m‐MDSCs and align with previous reports linking MDSC burden to AML disease status. More importantly, they extend current understanding by highlighting the BM niche—the primary site of leukemogenesis—as a critical compartment where immunosuppressive myeloid cells accumulate and may modulate both disease progression and therapeutic outcomes.

Monocytes, as key components of the innate immune system, play vital roles in host defense by participating in phagocytosis, tissue repair, antigen presentation, and the regulation of T‐cell differentiation. These cells exhibit considerable heterogeneity and can be classified into distinct subsets based on surface marker expression, each endowed with specialized immunological functions. Classical monocytes primarily contribute to phagocytosis and pathogen clearance, whereas intermediate monocytes are involved in antigen presentation, reactive oxygen species (ROS) production, and the induction of CD4^+^ T‐cell proliferation and differentiation [19, 20]. Shifts in monocyte subset distribution have been implicated in a range of pathological conditions, including cancer, infection, and autoimmune disorders [21]. Most studies have focused on the role of CD16^−^ and CD16^+^ monocyte subsets in malignant diseases and have shown that CD16^+^ monocyte cells can be a potential diagnostic disease and prognostic indicator, such as cholangiocarcinoma [22], colorectal cancer [23], and carcinoma [24]. In this study, we identified a significant increase in the proportion of intermediate monocytes within the BM monocyte cells of newly diagnosed AML patients compared to both HCs and those in CR. We acknowledge that the observed increase in total monocyte frequency among BM mononuclear cells following treatment could be partially attributed to the substantial depletion of the blast population, which would consequently increase the relative proportion of remaining cell types. To eliminate this potential confounding effect and better reflect the intrinsic shifts within the monocyte lineage, we specifically focused our analysis on the relative distribution of monocyte subsets within the total monocyte population rather than their frequency relative to all nucleated cells. Further longitudinal analysis with 1‐year follow‐up revealed that a higher percentage of intermediate monocytes served as an independent risk factor in both univariate and multivariate Cox regression models for survival, even after adjusting for ELN risk categories. The multivariate analysis demonstrated that both intermediate monocyte percentage (HR = 4.170, *p* = 0.034) and ELN 2022 risk classification (HR = 3.264, *p* = 0.019) maintained their independent prognostic value; the combination of monocyte subset analysis with established genetic risk stratification may provide a more comprehensive prognostic assessment, capturing both microenvironmental and genetic determinants of treatment outcome. The corresponding Kaplan–Meier analysis demonstrated that patients with elevated intermediate monocyte levels experienced significantly shorter OS and LFS. These findings suggest that intermediate monocytes may contribute to an immunosuppressive microenvironment that facilitates disease progression and relapse. Their association with adverse outcomes underscores their potential role not only as a prognostic biomarker but also as a mediator of AML pathogenesis. It will be essential to expand upon these findings by increasing the sample size in future investigations to strengthen the evidence.

To evaluate the systemic antitumor immune status of patients, this study further investigated lymphocyte subsets in peripheral blood. T lymphocyte subsets are key cellular components responsible for cellular immunity and immunoregulation. CD3^+^ represents total T lymphocytes, which include CD4^+^ helper T cells and CD8^+^ cytotoxic T cells. Numerous studies have indicated that the surveillance function of T lymphocytes plays a crucial role in the initiation, progression, and clearance of AML [25, 26]. In this study, we observed that the proportions of total T lymphocytes and CD8^+^ T cells in peripheral blood were negatively correlated with BM blast percentage. Additionally, we observed that while the absolute counts of both CD4^+^ and CD8^+^ T cells were significantly reduced in newly diagnosed AML patients compared to HCs, the CD4/CD8 ratio was notably elevated. This apparent paradox can be explained by the more profound reduction in CD8^+^ T cells, likely due to their heightened susceptibility to exhaustion in the tumor microenvironment, coupled with a relative expansion of immunosuppressive CD4^+^ T‐cell subsets, such as regulatory T cells (Tregs) [27]. This shift toward a higher CD4/CD8 ratio may represent an active immune evasion mechanism employed by AML. Following chemotherapy, we observed a progressive decrease in the CD4/CD8 ratio, which occurred irrespective of treatment response. This suggests that the ratio change may be more closely related to chemotherapy‐induced lymphodepletion and subsequent reconstitution dynamics rather than being a specific marker of therapeutic efficacy [28]. The preferential recovery of CD8^+^ T cells during immune reconstitution could be a key driver of this ratio decrease. In healthy individuals, the immune system continuously monitors and eliminates abnormal cells, including pre‐malignant clones. T and CD8^+^ cytotoxic cells serve as the primary effectors that directly kill abnormal cells such as blasts [29]. A high blast percentage reflects a highly malignant and proliferative tumor microenvironment, which can actively suppress or exhaust both total T and CD8^+^ T cells through various mechanisms. Furthermore, the proportion of intermediate monocytes was negatively correlated with the total T lymphocyte percentages, suggesting that intermediate monocytes may suppress the function and survival of anti‐tumor T lymphocytes. Collectively, by examining both the intrinsic tumor characteristics (blasts and monocyte subsets) and the systemic immune status (peripheral blood T cells), our findings provide convergent evidence for the central role of immune escape in the prognosis of AML.

## 5. Conclusion

Our findings indicate that BM intermediate monocyte subsets show promise as potential indicators of disease status in patients with AML. In our cohort, a higher proportion of intermediate monocytes was associated with adverse outcomes, including shorter OS and LFS, and remained significant in multivariate analysis. The observed negative correlations between intermediate monocytes and peripheral T cells, along with the inverse relationship between blast percentage and T‐cell immunity, suggest the possible involvement of intermediate monocytes in shaping an immunosuppressive microenvironment during disease progression. Additionally, our study detected elevated levels of m‐MDSCs among total monocytes in newly diagnosed AML patients compared to both HCs and CR patients, pointing to their potential role as markers of active disease, though they did not emerge as independent prognostic factors for survival. Collectively, these results highlight the potential utility of BM monocyte subset analysis while underscoring the complex interplay between innate and adaptive immunity in AML. This work provides a foundation for future studies to explore monocyte‐mediated immunosuppression as a potential therapeutic target.

## Ethics Statement

This study was approved by the Ethics Committee of the Zigong First People’s Hospital (Number 03202024).

## Disclosure

All authors have read and agreed to the published version of the manuscript.

## Conflicts of Interest

The authors declare no conflicts of interest.

## Author Contributions

Conceptualization: Yi Li and Chengjian Cao. Methodology: Liangjun Zhang and Yi Li. Formal analysis: Ziling Xu and Liangjun Zhang. Investigation: Huixiu Zhong. Writing – original draft preparation: Liangjun Zhang. Writing – review and editing: Yi Li and Liangjun Zhang. Visualization: Yi Li. Supervision: Minggang Yin and Jingyuan Huang. Project administration: Minggang Yin. Liangjun Zhang and Man Chen contributed equally to this work. Liangjun Zhang and Man Chen contributed equally to this work.

## Funding

This project was supported by the Zigong Key Science and Technology Plan (Collaborative Innovation Project of Zigong Academy of Medical Sciences) in 2023 (Grant 2023YKYXT01). Key Research and Development Science and Technology Program Project for High‐Quality Development 2024 of The First People’s Hospital of Zigong City (Grant 2024GZL03).

## Data Availability

The data supporting the findings of this study are available from the corresponding author upon reasonable request.
